# pH sensing via bicarbonate-regulated “soluble” adenylyl cyclase (sAC)

**DOI:** 10.3389/fphys.2013.00343

**Published:** 2013-11-25

**Authors:** Nawreen Rahman, Jochen Buck, Lonny R. Levin

**Affiliations:** Department of Pharmacology, Weill Cornell Medical CollegeNew York, NY, USA

**Keywords:** cyclic AMP, soluble adenylyl cyclase, bicarbonate, carbonic anhydrase, pH

## Abstract

Soluble adenylyl cyclase (sAC) is a source of the second messenger cyclic adenosine 3′, 5′ monophosphate (cAMP). sAC is directly regulated by bicarbonate (HCO^−^_3_) ions. In living cells, HCO^−^_3_ ions are in nearly instantaneous equilibrium with carbon dioxide (CO_2_) and pH due to the ubiquitous presence of carbonic anhydrases. Numerous biological processes are regulated by CO_2_, HCO^−^_3_, and/or pH, and in a number of these, sAC has been shown to function as a physiological CO_2_/HCO_3_/pH sensor. In this review, we detail the known pH sensing functions of sAC, and we discuss two highly-studied, pH-dependent pathways in which sAC might play a role.

Almost 40 years ago, pioneering work of Theodor Braun described a novel, “soluble” adenylyl cyclase (AC) from rat testis (Braun and Dods, 1975). From the initial reports, it became clear that this cyclase was unique; unlike the more widely studied, G protein-regulated transmembrane adenylyl cyclases (tmACs), soluble AC activity was not associated with the plasma membrane (Braun and Dods, 1975); was predicted to be approximately 48 kDa in size (Gordeladze et al., 1981), was hormone and heterotrimeric G protein insensitive (Braun et al., 1977), and its activity appeared to be dependent upon Mn^2+^-ATP as substrate (Braun and Dods, 1975). It was also shown that it was distinct from the previously identified soluble guanylyl cyclase (Braun et al., 1977; Neer and Murad, 1979). Despite extensive searching, soluble AC activity had been detected only in testis (Neer, 1978). A membrane-associated activity found in sperm was similarly hormone and G protein insensitive (Braun and Dods, 1975; Stengel and Hanoune, 1984; Garty and Salomon, 1987; Rojas and Bruzzone, 1992), and it was hypothesized that during maturation in the testis and/or during the transport of sperm through the epididymis, a single, novel form of AC would change from being cytosolic to membrane-attached (Braun and Dods, 1975). Subsequent studies confirmed sAC was responsible for soluble activity in testis and the majority of the membrane-associated activity in sperm (Esposito et al., 2004; Hess et al., 2005).

## Purification and cloning of sAC

Numerous attempts to purify this unique cAMP-producing enzyme failed. In 1999, starting from 950-rat testis, our laboratory purified a 48 kDa candidate protein band which eluted with soluble AC activity (Buck et al., 1999, 2002). The amino acid sequences of three peptides derived from this 48 kDa candidate protein revealed it represented a novel protein and were sufficient to isolate its cDNA by degenerate PCR (Buck et al., 1999). The initial screen of a rat testis cDNA library identified three different clones, the longest of which had an open reading frame predicting the full-length protein (sAC_fl_) would be 187 kDa. A second cDNA predicted a smaller, truncated sAC isoform, sAC_t_, which was subsequently shown to be a bona fide splice variant (Jaiswal and Conti, 2001), and which corresponds to the originally purified protein (Buck et al., 1999).

sAC_t_ comprises the amino terminal ~50 kDa of sAC_fl_, and it contains two domains (C1 and C2) homologous to the catalytic domains from other Class III nucleotidyl cyclases (Buck et al., 1999). Closely related catalytic domains are in ACs found in Cyanobacteria, and the biochemical properties of mammalian sAC (described below) are conserved from these bacterial orthologs (Steegborn et al., 2005a,b), which are thought to have evolved over 3 billion years ago.

Initial studies using the cloned sAC cDNA suggested it was abundantly expressed only in testis (Buck et al., 1999); however, subsequent studies using more sensitive RT-PCR (Sinclair et al., 2000; Farrell et al., 2008), mRNA microarrays (Geng et al., 2005), Western Blotting (Chen et al., 2000), or immunohistochemistry (Chen et al., 2013), revealed that sAC could be detected in nearly all tissues examined. Currently, sAC expression is considered to be ubiquitous. In individual cells, sAC can be found throughout the cytoplasm, inside or at various organelles and intracellular compartments—mitochondria, nuclei, microtubules, centrioles, mitotic spindles, and midbodies (Zippin et al., 2003). As such, sAC's intracellular localization provides an elegant solution to a long-standing conundrum inherent in the historical models for cAMP signaling. Prior to sAC's discovery and the demonstration that it is widely, if not ubiquitously expressed, and distributed throughout the cell, models for cAMP signaling were dependent upon cAMP production exclusively at the plasma membrane. These models required diffusion of the second messenger through the cytoplasm to its target effector proteins. Current models posit sAC localized at different cellular sites would provide specificity by transducing the cAMP signal exclusively to the appropriate player in close proximity to the cyclase (Wuttke et al., 2001; Zippin et al., 2003; Bundey and Insel, 2004).

## sAC activity

The early characterization of soluble AC activity revealed it was insensitive to the usual regulators of the more widely studied tmACs, including heterotrimeric G proteins (Braun and Dods, 1975) and the plant diterpene forskolin (Stengel et al., 1982; Forte et al., 1983); these properties were confirmed in the heterologously expressed sAC cDNAs (Buck et al., 1999). In partially purified preparations, soluble testis AC activity was reported to have a K_m_ for its substrate, ATP, of ~1–2 mM in the presence of the divalent cation Mn^2+^ (Gordeladze et al., 1981; Stengel and Hanoune, 1984; Rojas et al., 1993). Other divalent cations were found to support the enzymatic activity, but none supported activity to the same extent as Mn^2+^ (Braun, 1975). For example, in the presence of Mg^2+^, the enzyme's K_m_ for substrate ATP was reported to be above 15 mM (Stengel and Hanoune, 1984). In these early studies, there was no real consensus about regulation by Ca^2+^; in one report, Mn^2+^-ATP dependent activity was potentiated by Ca^2+^(Braun et al., 1977), but others found the same activity to be Ca^2+^ insensitive (Stengel and Hanoune, 1984). The membrane-associated, soluble AC-like activity found in sperm was thought to be stimulated by sodium bicarbonate (HCO^−^_3_) (Okamura et al., 1985; Garty and Salomon, 1987; Visconti et al., 1990), while in guinea pig sperm, Ca^2+^ activation was reported to be dependent upon HCO^−^_3_ (Garbers et al., 1982).

Heterologous expression of full-length (sAC_fl_) and truncated sAC (sAC_t_) isoforms revealed a significant difference in their specific activities (Buck et al., 1999; Jaiswal and Conti, 2003), which was subsequently shown to be due to an autoinhibitory domain present in the longer isoform (Chaloupka et al., 2006). It remains unclear how this autoinhibitory domain regulates sAC activity. sAC_fl_ also contains a unique Heme binding domain, but its contribution to activity also remains unclear (Middelhaufe et al., 2012). Despite these differences, sAC_t_ and sAC_fl_ share the same C1 and C2 catalytic domains (Buck et al., 1999), and much of their regulation is conserved (Buck et al., 1999; Chen et al., 2000; Chaloupka et al., 2006). Characterization of heterologously expressed and purified sAC_t_ confirmed many of the biochemical properties identified for purified soluble AC from testis, and they demonstrated that these properties were intrinsic to sAC. Purified sAC_t_ exhibits a K_m_ for substrate ATP of ~1 mM in the presence of Mn^2+^, and while its affinity for Mg^2+^ATP is in excess of 10 mM, Ca^2+^ stimulates the enzyme's activity by increasing its affinity for ATP to ~1 mM (Litvin et al., 2003). Its affinity for ATP is close to the concentration found in cells (Traut, 1994), prompting us to postulate that sAC may be sensitive to physiologically relevant changes in ATP (Litvin et al., 2003). We recently demonstrated this to be the case; sAC, but not tmAC, activity inside cells is sensitive to inhibitors which diminished ATP levels (Zippin et al., 2013). Cellular ATP levels increase in high glucose, and we showed that overexpression of sAC converts a cell line which normally does not alter cAMP levels in response to glucose into glucose-responsive cells. In addition, we found that in cells which are normally glucose responsive, pancreatic β cells, sAC is required for the ATP-dependent glucose response (Zippin et al., 2013). Other functions of sAC have been identified based upon phenotypes in sAC knockout mice (Esposito et al., 2004; Hess et al., 2005; Lee et al., 2011; Choi et al., 2012; Zippin et al., 2013) or by taking advantage of pharmacological inhibitors which distinguish between sAC and tmACs (Bitterman et al., 2013), including functions dependent upon cellular Ca^2+^. Tumor Necrosis Factor (TNF) (Han et al., 2005) and neurotrophin (NGF) signaling (Stessin et al., 2006) and glucose sensing (Ramos et al., 2008) are mediated via Ca^2+^-dependent regulation of sAC.

sAC is directly stimulated by the bicarbonate anion (HCO^−^_3_)(Chen et al., 2000). HCO^−^_3_ stimulates sAC via two mechanisms; it relieves substrate inhibition and elevates *V*_max_ (Litvin et al., 2003) by facilitating closure of the active site (Steegborn et al., 2005b). As previously predicted (Garbers et al., 1982), HCO^−^_3_stimulation is synergistic with Ca^2+^ (Litvin et al., 2003). HCO^−^_3_-dependent regulation of sAC plays a role in sperm activation (Esposito et al., 2004; Hess et al., 2005), activity-dependent metabolic communication between astrocytes and neurons (Choi et al., 2012), and multiple processes in the eye, including aqueous humor formation (Lee et al., 2011), retinal ganglion cell survival (Corredor et al., 2012) and corneal endothelial cell protection (Li et al., 2011).

Due to the ubiquitous presence of carbonic anhydrases (CAs), HCO^−^_3_regulation means that sAC activity inside cells will be modulated by changes in CO_2_ and/or pHi (Tresguerres et al., 2010a; Buck and Levin, 2011). CO_2_-dependent regulation of sAC plays a role inside the mitochondrial matrix, where metabolically generated CO_2_ modulates the synthesis of ATP by the electron transport chain via sAC-generated cAMP (Acin-Perez et al., 2009, 2011; Di Benedetto et al., 2013; Lefkimmiatis et al., 2013), and in airway cilia, where CO_2_ regulates the ciliary beat frequency via sAC (Schmid et al., 2007, 2010).

## sAC as pH sensor

Although its *in vitro* activity is insensitive to physiologically relevant pH changes (Chen et al., 2000), the pH-dependent equilibrium between CO_2_ and HCO^−^_3_ means that cellular sAC activity will be regulated by local changes in pH. In fact, sAC has been shown to play a role in pH dependent movements of the electrogenic, proton pumping vacuolar–ATPase (V-ATPase) in a number of physiological contexts (Pastor-Soler et al., 2003; Paunescu et al., 2008, 2010; Tresguerres et al., 2010b). In epididymis and proximal tubules of the kidney, V-ATPases translocate to the apical surface of the cell in response to a pH change in the corresponding lumen. sAC is in a complex with V-ATPase (Paunescu et al., 2008), and the V-ATPase translocation is mediated by sAC, in a CA dependent manner (Pastor-Soler et al., 2003, 2008). This pH dependent signaling is evolutionarily conserved; a similar mechanism, involving CA, sAC, and V-ATPase, is responsible for organismal pH homeostasis in shark (Tresguerres et al., 2010b). Interestingly, in shark gills the V-ATPase translocates to the basolateral side of the pH sensing cells. Thus, sAC-dependent V-ATPase mobilization is a conserved mechanism of pH sensing. The appreciation that pH sensing can be achieved via HCO^−^_3_ regulation of sAC instead of being exclusively dependent upon proton sensing suggests there may be additional pH-dependent physiological processes dependent upon sAC. We discuss two possible examples below.

## pH sensing in the endosomal-lysosomal pathway

The endo-lysosomal system is central to the processes of autophagy and endocytosis (Klionsky, 2007; Mizushima, 2007), and there is growing appreciation of its involvement in a broad range of diseases (Futerman and Van Meer, 2004; Nixon et al., 2008). As internalized materials pass from early to late endosomes and finally to lysosomes, the lumen of the endocytic organelles become more acidic. Lysosomes are the terminal compartment of both endocytic and autophagic pathways, and within lysosomes, acid hydrolase enzymes degrade proteins, lipids, and polysaccharides. The pH of the lysosome lumen is maintained between 4 and 5 (Pillay et al., 2002), which is the optimal pH for lysosomal enzyme activity.

Regulation of lysosomal pH is a complex process involving multiple channels and transporters. Acidification of lysosomes is accomplished by the electrogenic V-ATPase, which pumps protons into the lysosomal lumen (Forgac, 2007). Chloride movement through an opposite conductance pathway (Jentsch, 2007) (mediated at least in part via CLC7) and efflux of cations (Steinberg et al., 2010) facilitate vesicle acidification by neutralizing the positive charge and reducing the membrane potential caused by the pumped protons. Little is known about how the V-ATPase sets the pH or how these parallel ion transports are regulated. In particular, no pH-sensitive signaling cascades have been implicated.

Cyclic AMP has been shown to modulate lysosomal pH in macrophages (Di et al., 2006), microglia (Majumdar et al., 2007) and retinal pigment epithelium (RPE) cells (Liu et al., 2008). The cAMP effector, Protein Kinase A (PKA) increases chloride conductance (Bae and Verkman, 1990), possibly via the chloride channel CLC7. Lysosomal acidification in microglia is enhanced by upregulation of CLC7 (Majumdar et al., 2011), in what is thought to be a PKA dependent process (Majumdar et al., 2007). However, how the cAMP “second messenger” is made and whether cAMP levels are dependent upon pH remains unknown. It is tempting to postulate that sAC is the pH regulated source of cAMP regulating these processes.

Like lysosomes, both early and late endosomes are maintained within certain pH ranges; early endosomes range between pH ~5.9-6.8 whereas late endosomes range between pH ~4.9 and 6.0 (Maxfield and Yamashiro, 1987). Endosomal acidification is linked to intracellular trafficking, but it remains unknown how early endosomes “set” luminal pH to ~6.5 and late endosome/lysosomal set their luminal pH to ~5. Endosomal pH is maintained via similar proteins as control lysosomal pH, but endosomes use distinct isoforms of V-ATPases and chloride channels [for a complete review, see Forgac (2007), Stauber and Jentsch (2013)]. In such cases, different isoforms need to be trafficked to the endosomes or their activity modulated in order to establish and maintain the proper pH. sAC has already been shown to modulate the pH-dependent translocation of the V-ATPase to plasma membranes (Pastor-Soler et al., 2003; Tresguerres et al., 2010b); might sAC-generated cAMP play a role in trafficking V-ATPases or other chloride channels to endosomal/lysosomal membranes and hence establishing intra-vesicular pH?

## pH sensing in osteoclasts

Bone remodeling is a tightly regulated process involving bone formation by osteoblasts and bone resorption by osteoclasts. Imbalances in either result in bone diseases. For example, excessive resorption of bone due to increased activity or number of osteoclasts leads to osteoporosis, while deficient osteoclastic bone resorption leads to osteopetrosis (high bone mineral density) (Lazner et al., 1999; Qin et al., 2012). Bicarbonate and pH are known regulators of osteoclasts. Bone resorption is inhibited by high HCO^−^_3_ or alkaline pH and stimulated by low HCO^−^_3_ or acidic pH (Kraut et al., 1986; Goldhaber and Rabadjija, 1987; Arnett and Dempster, 1990; Krieger et al., 1992; Geng et al., 2009). *In vitro*, high concentrations of HCO^−^_3_ decreased osteoclast formation and growth. The effects of HCO^−^_3_ were blocked by inhibitors of sAC and recapitulated by addition of exogenous cAMP (Geng et al., 2009), suggesting sAC activity inhibits osteoclast formation. Consistent with this potential role, human sAC was identified as a candidate gene in the locus tightly associated with hyperabsorptive hypercalciuria (Reed et al., 1999), a disease characterized by low bone mineral density.

In osteoclasts, V-ATPase is an essential player in bone reabsorption. Acidification of the resorptive lacunae (to a pH of ~4.5) is achieved by the combined actions of the V-ATPase and CLC-7 clustered at high density in the ruffled border (Kornak et al., 2001; Qin et al., 2012). A continuous flow of H^+^ and Cl^−^ dissolves the mineral component of bone and enhance the activity of enzymes that digest the organic matrix. The important role played by the V-ATPase/CLC-7 complex suggests potential involvement of sAC, and might indicate that sAC contributes to multiple pH-dependent functions in bone.

## Conclusions

sAC functions as a pH sensor in numerous physiological systems. Historically, pH sensors were assumed to be proteins directly regulated by protons. With the discovery of sAC, it is clear that this dogma needs revision; pH sensing can also be achieved via a protein directly regulated by HCO^−^_3_.

### Conflict of interest statement

Levin and Buck own equity interest in CEP Biotech which has licensed commercialization of a panel of monoclonal antibodies directed against sAC.
